# Automatic Detection, Classification, and Grading of Lumbar Intervertebral Disc Degeneration Using an Artificial Neural Network Model

**DOI:** 10.3390/diagnostics13040663

**Published:** 2023-02-10

**Authors:** Wongthawat Liawrungrueang, Pyeoungkee Kim, Vit Kotheeranurak, Khanathip Jitpakdee, Peem Sarasombath

**Affiliations:** 1 Department of Orthopaedics, School of Medicine, University of Phayao, Phayao 56000, Thailand; 2 Department of Computer Engineering, Silla University, Busan 46958, Republic of Korea; 3 Department of Orthopaedics, Faculty of Medicine, King Chulalongkorn Memorial Hospital, Chulalongkorn University, Bangkok 10330, Thailand; 4 Center of Excellence in Biomechanics and Innovative Spine Surgery, Chulalongkorn University, Bangkok 10330, Thailand; 5 Department of Orthopedics, Queen Savang Vadhana Memorial Hospital, Chonburi 20110, Thailand; 6 Department of Orthopaedics, Faculty of Medicine, Chiang Mai University, Chiang Mai 50200, Thailand

**Keywords:** automation model, intervertebral disc degenerations, lumbar spine, MRI, deep learning, diagnostic performance, computer neural network

## Abstract

Background and Objectives: Intervertebral disc degeneration (IDD) is a common cause of symptomatic axial low back pain. Magnetic resonance imaging (MRI) is currently the standard for the investigation and diagnosis of IDD. Deep learning artificial intelligence models represent a potential tool for rapidly and automatically detecting and visualizing IDD. This study investigated the use of deep convolutional neural networks (CNNs) for the detection, classification, and grading of IDD. Methods: Sagittal images of 1000 IDD T2-weighted MRI images from 515 adult patients with symptomatic low back pain were separated into 800 MRI images using annotation techniques to create a training dataset (80%) and 200 MRI images to create a test dataset (20%). The training dataset was cleaned, labeled, and annotated by a radiologist. All lumbar discs were classified for disc degeneration based on the Pfirrmann grading system. The deep learning CNN model was used for training in detecting and grading IDD. The results of the training with the CNN model were verified by testing the grading of the dataset using an automatic model. Results: The training dataset of the sagittal intervertebral disc lumbar MRI images found 220 IDDs of grade I, 530 of grade II, 170 of grade III, 160 of grade IV, and 20 of grade V. The deep CNN model was able to detect and classify lumbar IDD with an accuracy of more than 95%. Conclusion: The deep CNN model can reliably automatically grade routine T2-weighted MRIs using the Pfirrmann grading system, providing a quick and efficient method for lumbar IDD classification.

## 1. Introduction

The incidence of low back pain varies widely in different reports. It is the fifth most common cause for a visit to the doctor and affects up to 40% of patients [1]. Low back pain is strongly connected to the degenerative process of intervertebral discs. The height of the affected intervertebral disc gradually falls, causing a change in the dynamics of the affected segment of the spine [2].

Intervertebral disc degeneration (IDD) is a common cause of symptomatic axial low back pain and can be caused by several factors. It also represents an important cause of morbidity and mortality. Numerous factors can initiate degenerative processes, which most commonly affect the nucleus pulposus and ultimately influence the biomechanics of the entire spine [3]. The number of individuals diagnosed with symptomatic lumbar disc herniation increases with age, and the incidence of these diseases is higher in women than in men [4]. The duration, severity, type, and position of loads affect the state of the intervertebral disc and, thus, the biological response to these factors [5]. The current reviews of symptomatic low back pain with degenerative changes show it to be a common problem in elderly patients with an orthopedic disease. Symptomatic degenerative disc disease represents between 30 and 40% of cases of degenerative spinal disease. Intervertebral discs are pads of fibrocartilage that resist spinal compression while permitting limited movement. Even when the spine is bent or extended, they distribute the weight equally across the vertebral bodies. The retention of damaged macromolecules is encouraged by degeneration, which likely results in decreased tissue strength [6].

In the United States, nearly USD 50 billion is spent annually for the evaluation and treatment of low back pain [7]. Magnetic resonance imaging (MRI) is currently the most commonly ordered test to evaluate patients with sciatica, and it is the standard for the investigation and diagnosis of IDD [8]. The high prevalence of asymptomatic intervertebral disc diseases results in a need for an accurate diagnosis, which is key to management [9].

### 1.1. Medical Image Modalities

**Figure 1:**
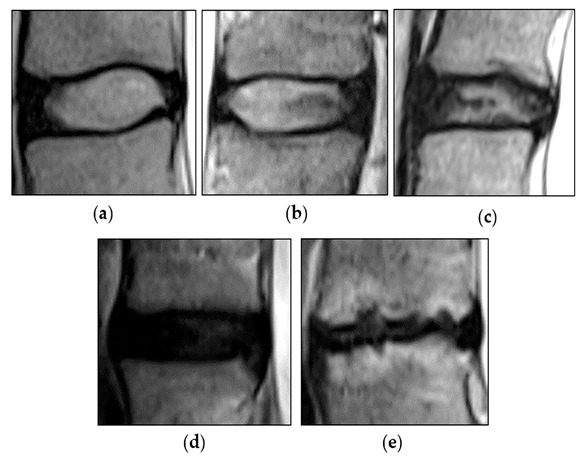
The Pfirrmann grading system: grade I (**a**), grade II (**b**), grade III (**c**), grade IV (**d**), and grade V (**e**).

Magnetic resonance imaging (MRI) is the standard imaging modality used to detect IDD. It has the advantage of being radiation-free and allowing for a multiplanar evaluation with good soft tissue contrast, allowing for a more accurate interpretation of disc changes [10]. The common MRI classification used by spine surgeons in the interpretation of degenerative disc disease and its related problems is the Pfirrmann classification for disc morphology, which classifies changes in disc degeneration progression in stages, indicating more progression in the destruction of the disc architecture as one moves from grade I to V [11] (Figure 1). 

While radiographs can provide useful information, especially for identifying intervertebral disc degeneration or calcification, there are notable limitations [12]. 

### 1.2. Convolutional Neural Network (CNN) for MR Image Analyses

In recent years, it has become natural to hypothesize that neural networks and deep learning methods can be harnessed for the effective detection of IDD in MRI sequences [7]. Machine learning and artificial intelligence (AI) are powerful tools with the ability to improve the understanding of predictive metrics in clinical spine surgery [13]. Many studies have shown that AI programs can be used to extract “radiomic” information from images that is not discernible by a visual inspection with the naked eye, potentially increasing the diagnostic and prognostic value derived from image datasets [14], as well as increasing the value that musculoskeletal imagers provide to patients and referring clinicians through improved image quality, patient centricity, imaging efficiency, and diagnostic accuracy [15]. Moreover, there are many other innovative applications of AI in various technical aspects of processes ranging from removing image artifacts, normalizing/harmonizing images, improving image quality, lowering radiation and contrast dose, and shortening the duration of imaging studies [16].

Deep learning models of artificial intelligence can potentially provide tools to automatically detect IDD and provide visuals with significant speed. The purpose of this study was to investigate the potential of using deep convolutional neural networks (CNNs) for the detection, classification, and grading of IDD.

AI technology might be a future game changer in the medical field, with the possibility of being applied in many clinical settings. This technology could be implemented in hospital radiological imaging systems to analyze the MRI data of unclarified spinal diseases. Another potential application is as an additional tool for physicians to use in making accurate early diagnoses of IDD, decreasing medical errors and personal bias, reducing the routine hospital work load, and creating a new approach to detecting and classifying lumbar intervertebral disc degeneration, as well as similar medical applications. The technology could also shrink the ambiguous zone when making diagnoses of IDD via MR imaging, which could also reduce the mortality caused by delayed management, decrease unnecessary investigations, and lower costs, as well as assisting physicians to make accurate diagnoses. 

One of the most effective target detection networks is You Only Look Once (YOLO). Directly from the input frame, the YOLO network can forecast class probabilities and bounding boxes in an evaluation. Each input image from the training set is divided into square grids by the YOLO network. The grid is utilized to detect an object when it is filled by the center of the target ground truth. Several bounding boxes and their confidence scores are projected for each grid. Unlike other detection networks, the YOLO network can predict class probabilities and bounding boxes and can provide an assessment directly from the input frame. However, YOLO detectors need to be taught using annotated datasets in order to attain the highest variability of the target [17]. 

Ramesh et al. reported the Yolov5 method to automatically identify, locate, and describe microsurgical instruments from intraoperatively recorded footage of neurosurgery, which achieved a high 93.2% mean average precision [18]. A study by Gromada et al. presented a combination of YOLOv5 with post-processing using classic image analyses. That study reported that the new system improves both the classification accuracy and the location of the identified object [19].

Combining MR imaging with machine learning could help physicians to identify management needs earlier while reducing the need for time-consuming and expensive investigative procedures. This could also aid both physicians with limited experience and primary physicians who are often faced with difficult diagnostic situations. For example, with the advances in medical imaging technology and artificial intelligence (AI), many computer-aided detection (CAD) systems have been developed to increase the performance of lesion detection [20]. 

Currently, there are many research studies on using the deep learning model to detect or predict the severity of degenerative intervertebral disc disease, but no study reports on real-time prediction using YOLOv5 detectors.

Our findings imply that a single thorough CNN is capable of automatically diagnosing a variety of various lumbar spine degenerative abnormalities. The CNN provides a high diagnostic accuracy for real-time intervertebral disc interpretation.

Our objective was to evaluate the diagnostic performance of a convolutional neural network (CNN)—YOLOv5—trained on multiple MRIs of the intervertebral disc degeneration of the lumbar spine to determine the accuracy of its interpretation.

## 2. Material and Methods 

Sagittal IDD T2-weighted MRI images were obtained from an open-access lumbar spine MRI dataset from Sudirman et al., which contains an anonymized clinical MRI study [21]. We affirm that all procedures performed in this study were in accordance with ethical standards and comply with the 1964 Helsinki Declaration. Patient identification data were not collected.

### 2.1. Protocol for MRI Recruitment

The sample MRIs were obtained as sagittal images of IDD T2-weighted MRIs of 1000 disc levels from 200 adult patients from a standard dataset of 515 adult patients with symptomatic low back pain [21]. Computerized randomization was used to choose the image dataset. The inclusion dataset of the sagittal images of IDD T2-weighted MRIs (1000 disc levels from 200 adult patients) included in this study was of degenerative disc disease, where the diagnosis had been confirmed by a musculoskeletal radiologist. The exclusion criteria were patients whose IDD T2-weighted MRIs images showed tumors, infection, inflammatory disorders, congenital diseases, or a fracture of the lumbar spine. Training and validation data were separated before data augmentation.

### 2.2. Computational Environment

Python 3.6 was used in the development of the model. The algorithms were trained and validated using Google Colaboratory. In this study, a personal computer (PC) with an Intel I7 8700 K 3.70 GHz processor, 32 GB DDR4 RAM, Nvidia GeForce GTX 1080 8 GB, Anaconda with Python, and TensorFlow was used. The latest Anaconda was downloaded from https://www.anaconda.com/products/distribution accessed on 1 July 2022. This study aimed to create a Conda and Deep Learning Conda environment. A PC usually has a graphic card and a graphics processing unit (GPU) environment that can be used for deep learning.

### 2.3. Image Preprocessing 

**Figure 2:**
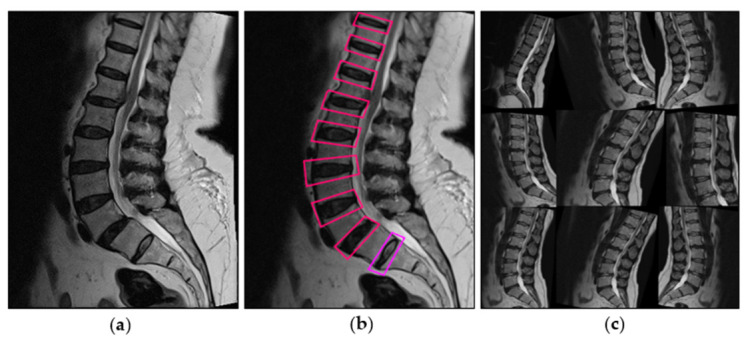
Dataset preparation: sagittal T2-weighted MRI of lumbar spine (**a**), annotation by a radiologist (**b**), image generation augmented by horizontal flip, cropping (zoom 0–20%), rotation (between −15° and 15°), shear (±15° horizontal and ±15° vertical) (**c**).

Focus slices of the 608 × 608 × 3 image were used to create a 304 × 304 × 12 image. Then, after the convolution of 32 kernels, a 304 × 304 × 32 feature map was obtained and processed. The region of interest was selected at the labeled lesion outlined by a musculoskeletal radiologist covering the adjacent upper and lower vertebral bodies and intervertebral discs in the mid-sagittal view (Figure 2). The images were cropped to 256 pixels on their shortest side. The images were then classified based on the Pfirrmann grading system [11] by a musculoskeletal radiologist.

### 2.4. Data Augmentation 

Because of the relatively small sample size, to prevent overfitting, all images were randomly augmented using the Python package Augmentor, which is a software package that focuses on offering functions that are frequently utilized in the creation of image data for machine learning, including flipping, rotating, zooming, scaling, cropping, and translating. In this study, we ran the standard image augmentation set with a horizontal flip, crop (zoom 0–20%), rotation (between −15° and 15°), and shear (±15° horizontal and ±15° vertical). The segmented images were shuffled and randomized for training. The final dataset contained 800 MRI images in the training dataset (80%) and 200 MRI images in the test dataset (20%). In each group, disc degeneration was classified based on the Pfirrmann grading system [11].

### 2.5. Deep Learning Training

**Figure 3:**
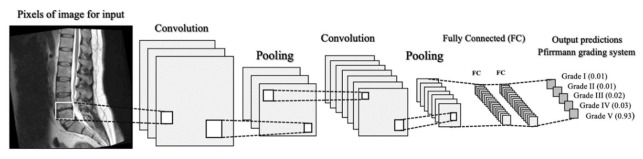
Deep learning architecture model used convolutional neural network (CNN).

**Figure 4:**
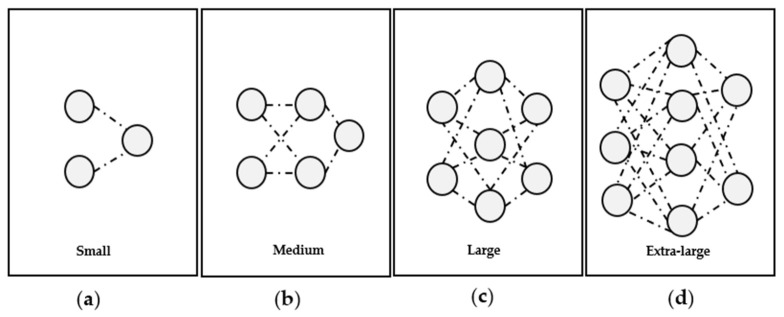
YOLOv5 architectural size of the neural network model: Small-YOLOv5s (**a**), Medium-YOLOv5m (**b**), Large-YOLOv5l (**c**), and Extra-large-YOLOv5x (**d**).

The deep learning method used the deep CNN model to train, detect, and grade the IDDs (Figure 3). However, this model cannot determine the segment of the spine images. The training CNN model was verified by testing the grading of the dataset using an automatic model. We used the YOLOv5 architecture (Figure 4) for high-performance object detection, which divides an image into a grid system, where each grid module detects objects within itself. The images can also be used for real-time object detection based on data streams, a process that requires very few computational resources. A total of 100 epochs were performed to test the model because the model’s accuracy increases until 100. 

### 2.6. Model Performance Evaluation

Accuracy, precision, recall, F1 score, and mean average precision were used to evaluate model performance. 

## 3. Results

**Figure 5:**
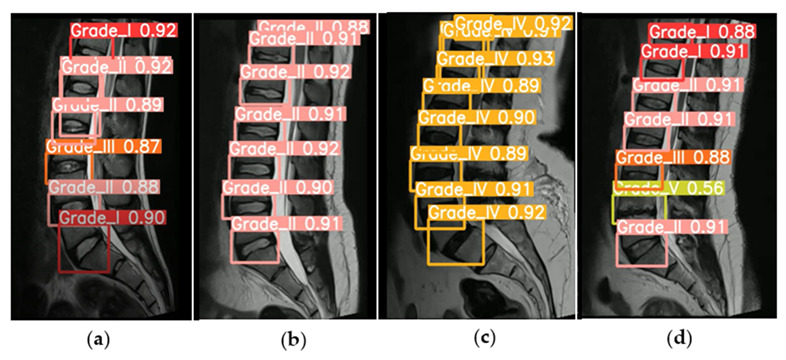
Results of automatic detection and grading of lumbar degenerative discs using convolutional neural network (CNN) with YOLOv5. Prediction based on the Pfirrmann grading system: patients with grade I–III (**a**), patients with grade II (**b**), patients with grade IV (**c**), and patients with grade I–V (**d**). The picture demonstrates the grading and the mean average precision.

We collected the sagittal intervertebral disc lumbar MRI training dataset from an open-resource dataset, which included a total of 1000 disc levels from 515 adult patients. The data were augmented and used to train and evaluate the models using the YOLOv5 model. Based on the Pfirrmann grading system, the results found 220 cases of grade I, 530 of grade II, 170 of grade III, 160 of grade IV, and 20 of grade V (Figure 5). 

**Figure 6:**
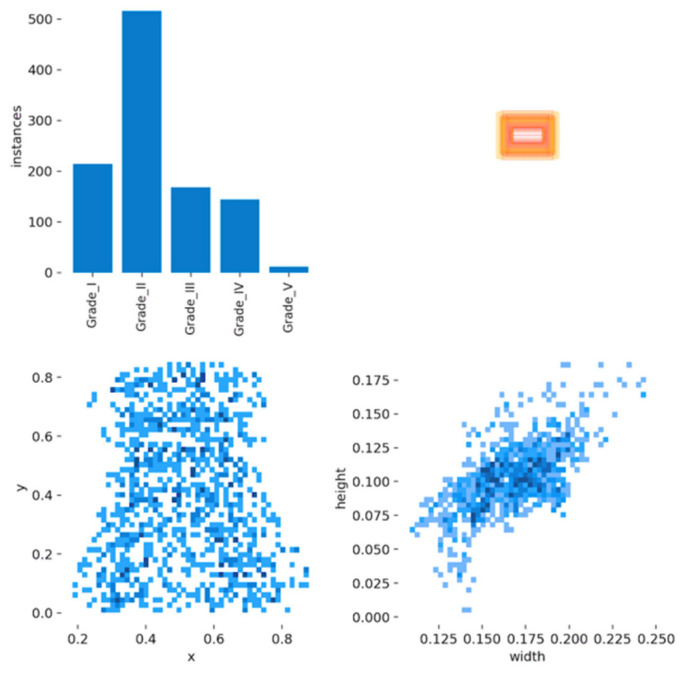
The IDD distribution of grades and shape information of bounding boxes of the dataset after using the augmentation technique.

The deep CNN model was able to detect and classify lumbar IDD with an accuracy of more than 95%. The training of the CNN model was verified using the test dataset and was found to have an automatic classification and detection of IDD accuracy of more than 95%. Figure 6 shows the IDD distribution of the grades and the shape information of the bounding boxes.

**Figure 7:**
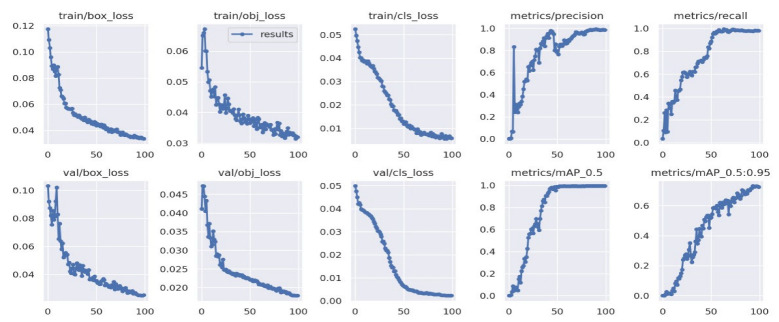
Performance training analysis plot graphs of the CNN network with a large YOLO-V5 model.

**Figure 8:**
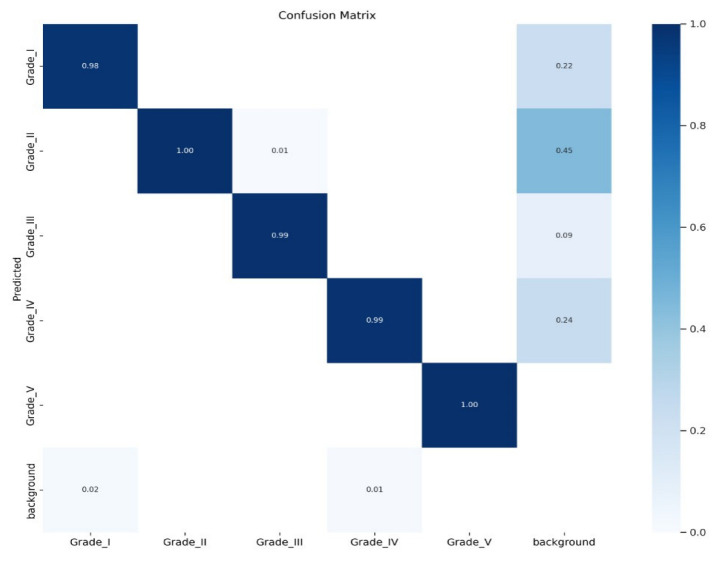
Confusion matrix representation and accuracy of the training model. (Grade I accuracy 0.98, grade II 1.0, grade III 0.99, grade IV 0.99, grade V 1.0). A column represents an instance of the actual class, whereas a row represents an instance of the predicted class.

Figure 7 shows the training results for the dataset. There are three types of losses: box_loss, obj_loss, and cls_loss. A box_loss represents the difference between the target disk box and the detected one. An obj_loss is the difference in object existence for each grid. cls_loss represents the misclassification of class between the target and detected objects. All loss values decreased sufficiently as the epoch numbers grew, showing that the training was successful. The precision and recall values were good, as they were close to one. The most frequently used metric, the mean average precision, was also satisfactory. While AP represents the average precision for each class, mAP shows the AP for all classes, and mAP_0.5 represents a mAP value under a fixed Intersection over Union (Boussios, #141) of 0.5. mAP_0.5:0.95 represents mAP values between IOU 0.5 and 0.95 intervals, where the step size is 0.05. All these graphs of the results show that the training was carried out well for the dataset under the default training hyper-parameters. Figure 8 shows the confusion matrix for the CNN applied to the collected data accuracy results for each grade of IDD, all of which were above 0.98. The light blue in the “background” is probably objects that should have been classified but were not.

**Figure 9:**
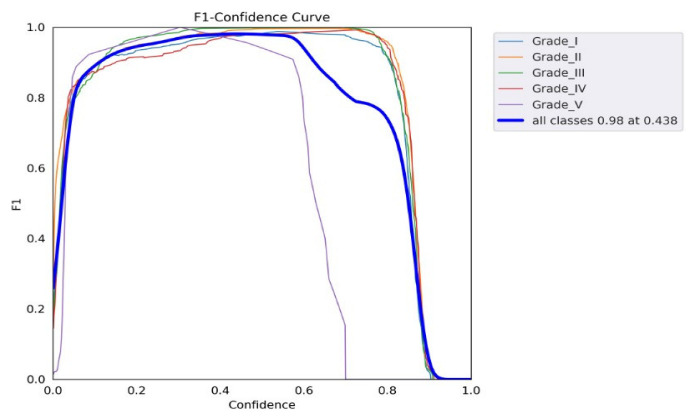
F1 score graph showing the relationship between F1 and the confidence curve.

Figure 9 shows the F1 scores for the confidence values, where confidence is defined as the recognition reliability for each detected IDD box. The F1 score shows the relative recognition performance between the precision and recall values. The figure shows the IDD F1 scores for all grades, where the best is 0.98 at a confidence level of 0.438. 

**Figure 10:**
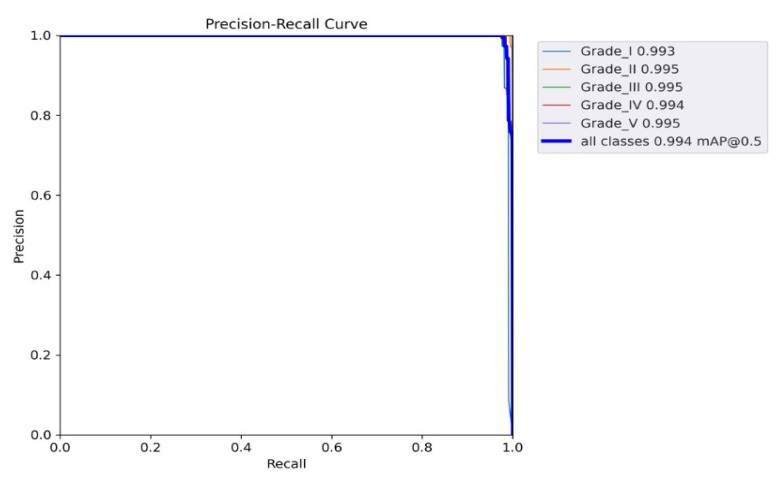
Recall–precision graph showing the relationship between recall and precision.

**Figure 11:**
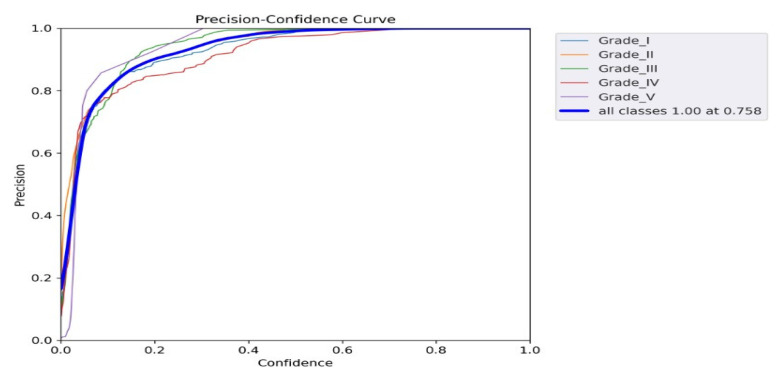
Precision–confidence graph showing the relationship between precision and confidence.

**Figure 12:**
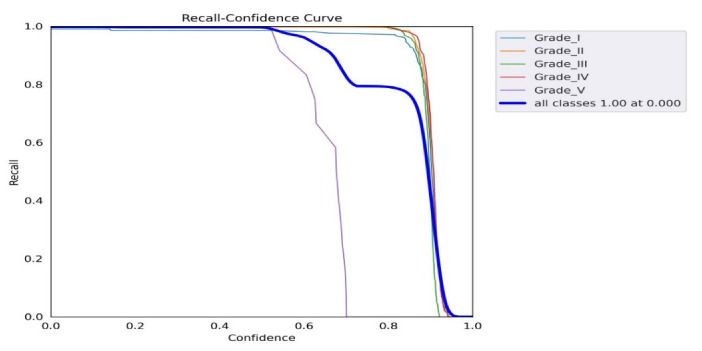
F1 score graph showing the relationship between the F1 and confidence curves.

Figure 10 shows the precision–recall curve; the graph’s lower left area was used for the AP score calculation. The precision vs. confidence values for all grades of IDD are shown in Figure 11, where a confidence level above 0.758 indicates satisfactory score precision. A similar relationship between the confidence and recall values is shown in Figure 12.

## 4. Discussion

Understanding the risks, prevention, treatments, and possible outcomes of spine injuries, as well as the incidence of degenerative spinal pathologies and pertinent demographic risk factors, is crucial for separating acute injuries from degenerative pathologies.

The gold standard for diagnosis and investigation is currently radiographic MRI imaging for classification and grading that can indicate prognosis and inform doctors’ decision making regarding management (non-operative or operative treatment) based on the Pfirrmann grading system, which is widely used by radiologists and orthopedists. However, that process is time-consuming for radiologists who are already overloaded with work related to prediction and having to classify the level of each patient. Moreover, 70% of all medical errors in diagnostic radiography are “missed findings” according to an examination of those errors [22]. This significant error rate demonstrates how difficult the “detection process” is for humans.

The application of artificial intelligence (AI) technology to medical imaging is growing. The potential of AI for practical applications has been unlocked by advances in machine learning (ML) and deep learning (DL), which facilitate procedures requiring high levels of cognitive ability, increase the accuracy of radiologist diagnoses, and have many more applications [23]. The many benefits and advantages of AI technology represent a future trend in computer-assisted diagnoses for improving the screening and detection of pathology lesions that will help the health care system. The Cross-ministerial Strategic Innovation Promotion Program (SIP) “Innovative AI Hospital System” project supported by the Japanese government emphasizes that it is essential that healthcare professionals and the general public share diverse updated and useful information in order to maintain or improve the quality of health and medical care systems [24].

In 2020, a study found that one of the many ways to succeed in training the convolutional neural network was to use a convolutional neural network called “VGG16” to correctly detect intervertebral disc lesions. That study, however, did not use the Pfirrmann grading system. The objective of the present study was to develop machine learning or deep learning algorithms that can detect, classify, and grade IDD. This study is the first to use a CNN with the YOLO-v5 model to detect degenerative disc disease and classify the severity into five grades based on the Pfirrmann grading system with a high accuracy. The advantage of the YOLO-v5 model includes the use of real-time detection systems, such as the VDO file and monitoring screen detection. We used this model to detect IDD in our routine work as an optional clinical application to assist orthopedists at our center. We also demonstrated the real-time detection and grading of degenerative disc disease using the YOLO-v5 model (Video S1).

The YOLO model is a one-stage detection model. The mean average precision is the current benchmark metric used by the computer vision research community to evaluate the robustness and accuracy of an object detection model [25] and represents an essential parameter for network model training. From the F1 curve, it was found that the confidence value that optimized the precision and recall was 0.438. In many cases, a higher confidence value is desirable. In the case of this model, it may be optimal to select a confidence of 0.6 since the F1 value appeared to be about 0.97, which is not far off from the maximum value of 0.98. Observing the precision and recall values at a confidence of 0.6 also confirms that this may be a suitable design point. Starting at about 0.6, the recall value began to decline, and the precision value still remained roughly at the maximum value (Figure 9). Based on our findings, we may apply this result setting to the other dataset to prove and find the most accurate point in order to improve real-time, better personalized, population medicine at lower costs and optimize decision making. It will become simpler to classify problems according to which solution strategy is the most rational as machine learning researchers and practitioners gather more experience. The acceptance of the addition of such systems is anticipated to increase as enough high-impact software systems based on mathematics, computer science, physics, and engineering are incorporated into clinics’ daily workflows.

A limitation of this study is that we used an open assessment dataset of MRIs of symptomatic low back pain that was imbalanced and varied in the distribution of data, as evidenced by the fact that grades IV and V were rarely found and that grades I and II were the most common types [26]. Due to the use of a provided open assessment dataset of MRIs, the data were anonymized, so they did not report on the identification of the participants, the relevant dates, the periods of recruitment, the eligibility criteria, the sources and methods of selection, exposure, follow-up, and data collection [21]. A radiologist was the only person who classified the photos using the Pfirrmann Classification. If two radiologists classify the same set of data, deviations can happen [27]. This model focuses only on the severity grading of the IDD. It cannot determine the segment of the spinal images. This study introduced a novel technique of using machine learning to simultaneously identify and differentiate types of spinal cord disorders. The local image gradient orientation properties of each structure were determined using machine learning. K-fold Cross-Validation is the preferred method to evaluate model performance, although the YoloV5 model can report the Model mAP, recall, and F1 score. The data cannot be generalized to all patients, as the dataset contains anonymized clinical MRI studies and, thus, does not provide an accurate description of the dataset. Additionally, a larger and a multi-center dataset would be desirable for the training, validation, and testing of advanced deep learning in the future. However, this is a standard public dataset, and this study was completely operated by a computer without human bias, so there were no sources of bias.

## 5. Conclusions

The new system of the automatic detection and classification of lumbar intervertebral disc degeneration grading described in this study represents a step forward in the computer-aided diagnosis and image-guided evaluation of spine diseases. This study demonstrates that a deep learning model can accurately classify and grade IDDs of the spine. A deep CNN model can automatically and reliably grade routine T2-weighted MRIs using the Pfirrmann grading system, providing a quick and efficient method for lumbar IDD classification. This method is practical for use in orthopedic and neurological applications, as well as for disease and anomaly identification. Continued development of the model using a larger, multi-center dataset is recommended.

## Data Availability

The data used in this research were acquired from a public resource.

## Supplementary Materials

The following supporting information can be downloaded at https://www.mdpi.com/article/10.3390/diagnostics13040663/s1, Video S1: Real-time detection and grading of degenerative disc disease using YOLO-v5 model.

## References

[1] Hall J.A., Konstantinou K., Lewis M., Oppong R., Ogollah R., Jowett S. (2019). Systematic review of decision analytic modelling in economic evaluations of low back pain and sciatica. Appl. Health Econ. Health Policy.

[2] Boxberger J.I., Orlansky A.S., Sen S., Elliott D.M. (2009). Reduced nucleus pulposus glycosaminoglycan content alters intervertebral disc dynamic viscoelastic mechanics. J. Biomech..

[3] Kos N., Gradisnik L., Velnar T. (2019). A Brief Review of the Degenerative Intervertebral Disc Disease. Med. Arch..

[4] Kim Y.K., Kang D., Lee I., Kim S.Y. (2018). Differences in the Incidence of Symptomatic Cervical and Lumbar Disc Herniation According to Age, Sex and National Health Insurance Eligibility: A Pilot Study on the Disease’s Association with Work. Int. J. Environ. Res. Public Health.

[5] Hanımoğlu H., Cevik S., Yılmaz H., Kaplan A., Çalış F., Katar S., Evran Ş., Akkaya E., Karaca O. (2019). Effects of Modic type 1 changes in the vertebrae on low back pain. World Neurosurg..

[6] Adams M.A., Roughley P.J. (2006). What is intervertebral disc degeneration, and what causes it?. Spine.

[7] Oktay A.B., Albayrak N.B., Akgul Y.S. (2014). Computer aided diagnosis of degenerative intervertebral disc diseases from lumbar MR images. Comput. Med. Imaging Graph..

[8] Amin R.M., Andrade N.S., Neuman B.J. (2017). Lumbar disc herniation. Curr. Rev. Musculoskelet. Med..

[9] Wu P.H., Kim H.S., Jang I.T. (2020). Intervertebral Disc Diseases PART 2: A Review of the Current Diagnostic and Treatment Strategies for Intervertebral Disc Disease. Int. J. Mol. Sci..

[10] Suthar P., Patel R., Mehta C., Patel N. (2015). MRI evaluation of lumbar disc degenerative disease. J. Clin. Diagn. Res. JCDR.

[11] Pfirrmann C.W., Metzdorf A., Zanetti M., Hodler J., Boos N. (2001). Magnetic resonance classification of lumbar intervertebral disc degeneration. Spine.

[12] da Costa R.C., De Decker S., Lewis M.J., Volk H. (2020). Diagnostic Imaging in Intervertebral Disc Disease. Front. Vet. Sci..

[13] Hopkins B.S., Yamaguchi J.T., Garcia R., Kesavabhotla K., Weiss H., Hsu W.K., Smith Z.A., Dahdaleh N.S. (2019). Using machine learning to predict 30-day readmissions after posterior lumbar fusion: An NSQIP study involving 23,264 patients. J. Neurosurg. Spine.

[14] Tang X. (2020). The role of artificial intelligence in medical imaging research. BJR Open.

[15] Gyftopoulos S., Lin D., Knoll F., Doshi A.M., Rodrigues T.C., Recht M.P. (2019). Artificial Intelligence in Musculoskeletal Imaging: Current Status and Future Directions. AJR Am. J. Roentgenol..

[16] Zhu G., Jiang B., Tong L., Xie Y., Zaharchuk G., Wintermark M. (2019). Applications of Deep Learning to Neuro-Imaging Techniques. Front. Neurol..

[17] Liu Q., Li Y., Dong Q., Ye F. (2022). Scene-Specialized Multitarget Detector with an SMC-PHD Filter and a YOLO Network. Comput. Intell. Neurosci..

[18] Ramesh A., Beniwal M., Uppar A.M., Vikas V., Rao M. Microsurgical Tool Detection and Characterization in Intra-operative Neurosurgical Videos. Proceedings of the 2021 43rd Annual International Conference of the IEEE Engineering in Medicine & Biology Society (EMBC).

[19] Gromada K., Siemiątkowska B., Stecz W., Płochocki K., Woźniak K. (2022). Real-Time Object Detection and Classification by UAV Equipped With SAR. Sensors.

[20] Obermeyer Z., Emanuel E.J. (2016). Predicting the Future—Big Data, Machine Learning, and Clinical Medicine. N. Engl. J. Med..

[21] Sudirman S., Al Kafri A., Natalia F., Meidia H., Afriliana N., Al-Rashdan W., Bashtawi M., Al-Jumaily M. (2019). Lumbar Spine MRI Dataset. https://data.mendeley.com/datasets/k57fr854j2/2.

[22] Kim Y.W., Mansfield L.T. (2014). Fool me twice: Delayed diagnoses in radiology with emphasis on perpetuated errors. AJR Am. J. Roentgenol..

[23] Yousefirizi F., Decazes P., Amyar A., Ruan S., Saboury B., Rahmim A. (2022). AI-Based Detection, Classification and Prediction/Prognosis in Medical Imaging. PET Clin..

[24] Nakamura Y. (2022). Japanese Cross-ministerial Strategic Innovation Promotion Program “Innovative AI Hospital System”; How Will the 4th Industrial Revolution Affect Our Health and Medical Care System?. JMA J..

[25] Bhattacharya A., Cloutier S.G. (2022). End-to-end deep learning framework for printed circuit board manufacturing defect classification. Sci. Rep..

[26] Shakeri A., Shakeri M., Ojaghzadeh Behrooz M., Behzadmehr R., Ostadi Z., Fouladi D.F. (2018). Infrarenal aortic diameter, aortoiliac bifurcation level and lumbar disc degenerative changes: A cross-sectional MR study. Eur. Spine J..

[27] Urrutia J., Besa P., Campos M., Cikutovic P., Cabezon M., Molina M., Cruz J.P. (2016). The Pfirrmann classification of lumbar intervertebral disc degeneration: An independent inter- and intra-observer agreement assessment. Eur. Spine J..

